# Human Antimicrobial Peptide Hepcidin 25-Induced Apoptosis in *Candida albicans*

**DOI:** 10.3390/microorganisms8040585

**Published:** 2020-04-17

**Authors:** Ruei-Ching Chen, Chung-Yu Lan

**Affiliations:** 1 Institute of Molecular and Cellular Biology, National Tsing Hua University, Hsinchu 30013, Taiwan; a104080818@gapp.nthu.edu.tw; 2 Department of Life Science, National Tsing Hua University, Hsinchu 30013, Taiwan

**Keywords:** *C. albicans*, antimicrobial peptide, hepcidin-25, apoptosis

## Abstract

Hepcidin 25 (hep 25) is a cysteine-rich 25-amino acid antimicrobial peptide containing the amino-terminal Cu(II)/Ni(II)-binding (ATCUN) motif. Upon metal binding, the ATCUN motif is known to be involved in the generation of reactive oxygen species (ROS), especially hydrogen peroxide and hydroxyl radicals, which act against different bacterial species. However, the antifungal activity and its correlation to the Cu(II)-ATCUN complex of Hep 25 are still poorly understood. Here, we found that ROS accumulation plays an important role in the fungicidal activity of hep 25 against *Candida albicans*. In addition, Annexin V-FITC staining and TUNEL assay results provide clues about the apoptosis induced by hep 25. Moreover, hep 25 also increases the generation of ROS, possibly because of copper binding to the ATCUN motif, which is relevant to its activity against *C. albicans*. Finally, the *C. albicans* killing action of hep 25 is an energy- and temperature-dependent process that does not involve targeting the membrane. Taken together, our results provide new insights into the mechanisms of hep 25 against *C. albicans* cells and the potential use of hep 25 and its derivatives as novel antifungal agents.

## 1. Introduction

The *Candida albicans* fungus is not only a member of the normal microbiota of healthy individuals, but also an opportunistic human pathogen [1,2]. Under normal conditions, *C. albicans* commensally colonizes the skin and mucosal surfaces without causing harm. However, it can become pathogenic and cause various clinical manifestations, including life-threatening systemic infection with a high mortality rate, particularly in immunocompromised patients [3,4]. Moreover, the antifungal drug resistance of *C. albicans* is also an expanding problem in clinical settings. Nowadays, only few classes of antifungals are currently available to treat *C. albicans* infections. Therefore, it is urgently required to develop new effective antifungal drugs.

Antimicrobial peptides (AMPs) play crucial roles in human innate immunity to combat microbial infections [5,6]. Additionally, human AMPs also contribute to diverse biological processes such as cell proliferation, wound healing and immune modulation [7,8]. Most AMPs comprise fewer than 100 amino acid residues and are positively charged and amphipathic [9]. AMPs are produced not only in humans but also in a wide variety of organisms, including bacteria, insects and plants [10]. To date, more than 3000 AMPs have been identified and cataloged in the antimicrobial peptide database (http://aps.unmc.edu/AP/), and the numbers of newly identified AMPs is increasing [11,12]. Because of a broad spectrum of targeted microorganisms, multiple mechanisms of action and relatively resistant variants, AMPs have the potential for therapeutic application. Several human AMPs are currently being tested in clinical trials, including LL37, PAC-113 (a derivative of salivary histatin 3) and PXL01 (a derivative of lactoferricin) [11,13,14].

Hepcidin 25 (hep 25) is a 25-amino acid β-defensin-like peptide that was first discovered in human urine and blood ultrafiltrate and found to be synthesized in the liver [15]. Hep 25 contains eight cysteine residues that form four disulfide bridges important for maintaining correct peptide folding and stabilizing the β-hairpin structure. Hep 25 is highly conserved in vertebrate species, particularly the eight cysteine residues and the N-terminal region containing the ATCUN motif [16,17]. Importantly, the ATCUN motif (H2N-X-X-H, where X is variable) is capable of specifically binding Cu(II) and Ni(II) metals. Moreover, the N-terminus of hep 25 is also critical for the interaction with the sole iron transporter, ferroportin (Fpn), to trigger internalization and degradation of Fpn, thereby acquiring nutritional immunity that limits pathogenicity during infection [18,19,20]. In addition to a master of iron regulation in the human body, Hep 25 is also an AMP with broad antimicrobial activity against gram-positive bacteria (e.g., *Staphylococcus aureus*, *Staphylococcus epidermidis*, *Enterococcus faecium*), gram-negative bacteria (e.g., *Pseudomonas aeruginosa, Escherichia coli*, *Acinetobacter baumannii*) and fungi (e.g., *C*. *albicans* and *Saccharomyces cerevisiate*) without causing cytotoxicity in mammalian cells [15,21,22,23,24]. Importantly, the bactericidal activity of hep 25 is dependent on the integrity of the disulfide bridges and correct folding of hep 25 [25]. Copper(II) chloride (CuCl_2_) can slightly enhance the bactericidal activity associated with the ATCUN motif [22]. Enhancing the bactericidal activity of hep 25 in acidic conditions has also been reported because the protonation states of its histidine residues increase the net positive charge of the peptide [22,26]. Although many studies have focused on the action of hep 25 against bacteria, the killing mechanism of hep 25 against fungal pathogens remains unknown.

In this study, we investigated the mechanism of hep 25 against *C. albicans* and showed that hep 25-mediated *C. albicans* killing is an energy- and temperature-dependent process without membrane lysis, making it similar to the mechanism of cell-penetrating peptides (CPPs). Moreover, the generation of reactive oxygen species (ROS) is coincident with the candidacidal activity of hep 25, and the copper-ATCUN complex of hep 25 is possibly the major driver of ROS production. Finally, hep 25 induced cell apoptosis of *C. albicans* cells. To our knowledge, this is the first report showing that hep 25 can cause apoptosis of a fungal pathogen cells and cross the membrane in a nonlytic manner.

## 2. Materials and Methods

### 2.1. Peptides and Regents

Hep 25 (H-Asp-Thr-His-Phe-Pro-Ile-Cys-Ile-Phe-Cys-Cys-Gly Cys-Cys-His-Arg-Ser-Lys-Cys- Gly Met-Cys-Cys-Lys-Thr-OH trifluoroacetate salt) was purchased from Bachem AG (Bubendorf, Switzerland). High-performance chromatography (HPLC) analysis showed that purity of the peptide was >90%. A stock solution of hep 25 was prepared in sterile double distilled water (ddH_2_O) and stored at −20 °C. All reagents were obtained from Sigma-Aldrich (St. Louis, MO, USA) unless indicated otherwise.

### 2.2. C. albicans Strain and Growth Conditions

The *C. albicans* SC5314 strain [27] was used throughout this study. Cells were plated on YPD agar (1% yeast extract, 2% Bacto peptone, 2% glucose and 1.5% agar) and grown overnight at 30 °C before each experiment. One colony was inoculated into 5 mL of YPD broth and incubated overnight (~14 to 16 h) at 30 °C with sharking at 180 rpm. Cells were harvested by centrifugation, washed twice with sterile sodium phosphate buffer (SPB), subcultured in YPD medium (OD600 = 0.2), and continuously grown to the exponential phase.

### 2.3. Calcein Leakage Assay

The effect of hep 25 on the integrity of the *C. albicans* plasma membrane was determined by measuring the leakage of calcein from small unilamellar liposome vesicles (SUVs, 100 nm diameter). The SUVs were composed of phosphatidylcholine (PC), phosphatidylethanolamine (PE), phosphatidylinositol (PI) and ergosterol in a ratio of 5:4:1:2 (*w/w/w/w*) and were prepared as previously described [28] with some modifications. The SUVs were treated with various concentrations (0, 6.25, 12.5, 25 and 50 μg/mL) of hep 25 at room temperature (RT). To evaluate pore formation and membrane integrity, a Cary Eclipse Fluorescence Spectrophotometer (PerkinElmer, Santa Clara, CA, USA) was used to measure the leakage of the calcein dye at excitation (λex) and emission (λem) of 490 and 535 nm, respectively. The percentage of dye-leakage was calculated as follows: % dye leakage = 100 × [(F − F0)/(Ft − F0)], where F is the fluorescence intensity after the peptide treatment, Ft is the fluorescence intensity with Triton X-100, and F0 is the fluorescence intensity of the untreated control SUVs. PC, PE and PI were purchased from Avanti Polar Lipids (Alabaster, AL, USA).

### 2.4. C. albicans Killing Assay

The antifungal susceptibility assay was performed as previously by colony-forming-unit (CFU) counting and propidium iodide (PI) staining as previously described [29] with some modifications. After growing overnight, *C. albicans* cells were subcultured in YPD, and grown at 30 °C for another 4.5 h. After collection by centrifugation, cell pellets were washed twice with 10 mM sterile sodium phosphate buffer (SPB, pH 5.8) and resuspended in SPB to 2.4 × 10^7^ cells/mL. Different concentrations of hep 25 were added to the cells, which were then incubated at 37 °C for 90 min. Each sample was serially diluted 10-fold, plated on YPD agar and incubated overnight at 30 °C. Cell viability was assessed by the number of CFUs. For PI staining, cells were treated with or without different concentrations of hep 25 as described above and harvested by centrifugation. After washing twice with phosphate-buffered saline (PBS), cells were resuspended in PBS containing 5 μg/mL PI. A flow cytometer (BD Accuri^®^ C6, BD Biosciences, San Jose, CA, USA) with the FL2 filter was used to measure the fluorescence intensity of PI-positive cells. Data analysis was performed using the BD Accuri^®^ C6 software (Version 1.0.26421) and discrimination of living cells and cell debris was based on forward versus side scatter (FSC vs. SSC). For each assay, 10,000 cells were analyzed, and the percentage of the PI-positive cells was displayed. Cells without hep 25 treatment were used as negative controls. All the experiments were repeated at least three times and the average fluorescence intensity was determined.

### 2.5. Analysis of Apoptotic Markers

Annexin V-FITC and propidium iodide (PI) staining was performed as previously described [30] with some modifications. Briefly, *C. albicans* cells were treated with various concentrations of hep 25 at 37 °C for 90 min. The cells were harvested by centrifugation, washed twice with phosphate-buffered saline (PBS), resuspended in 0.1 M potassium phosphate buffer (PPB, pH 7.2) containing 0.02 mg/mL Zymolyase 20T (Seikagaku, Inc., Tokyo, Japan) and sorbitol (at a final concentration of 2.4 M) at 30 °C for 10 min [31]. To asses externalization of phosphatidylserine (PS), the resulting protoplasts were stained with PI and FITC-labeled Annexin-V using the Annexin V-FITC/PI Apoptosis Detection Kit (Elabscience, Catalog #E-CK-A211, Huston, TX, USA) according to the manufacturer’s instruction. The stained cells were analyzed using a flow cytometer (BD Accuri^®^ C6, BD Biosciences). For each assay, 20,000 cells were analyzed and the percentage of the Annexin V-FITC positive and PI positive/negative cells in total cell population was displayed.

Terminal deoxynucleotidyl transferase dUTP nick-end labeling (TUNEL) (Roche, Catalog # 11684795910, Indianapolis, IN, USA) was performed as previously described [32] with some modifications. *C. albicans* cells were treated with various concentrations of hep 25 at 37 °C for 90 min. The cells were harvested by centrifugation, washed twice with PBS and fixed with 4% PFA at 30 °C for 15 min. The samples were then washed twice with PBS and incubated with 0.1 M PPB (pH 7.2) containing 0.02 mg/mL Zymolyase 20T and sorbitol (at a final concentration of 2.4 M) at 30 °C for 10 min. After washing twice with PBS, permeabilization solution (0.1 M sodium citrate [pH 6.0], with 0.1% Triton X-100) was added to the sample, incubated on ice for 2 min and washed again with PBS. Thereafter, fluorescein from an in situ cell death detection kit was used according to the manufacturer’s instructions (Roche Diagnostics GmbH, Mannheim, Germany), and the cells were examined using a confocal microscope (ZEISS LSM 800, Carl Zeiss, Jena, Germany).

### 2.6. Cytosolic ROS Assay

To detect intracellular ROS accumulation and hydroxyl radical production, dihydroethidium (DHE) and hydroxyphenyl fluorescein (HPF) (Cayman Chemical) fluorescent dyes were used, respectively [28,31]. *C. albicans* cells were treated with various concentrations of hep 25, 10 mM hydrogen peroxide (H_2_O_2_) and 1 mM bathocuproinedisulfonic acid (BCS) at 37 °C for 90 min. The cells were harvested, washed twice with PBS and stained with 20 µg/mL DHE or 5 μM HPF. The cells were then analyzed using a flow cytometer (BD Accuri^®^ C6, BD Biosciences) withthe FL1 filter (for HFP) and FL2 filter (for DHE), respectively.

### 2.7. Detection of Mitochondrial Membrane Potential (ΔΨm)

Mitochondrial function was assessed using 3,3′-dihexyloxacarbocyanine iodide (DiOC6(3); Molecular Probes) and tetramethylrhodamine (TMRM; Thermo Fisher), which are cell-permeant fluorescent dyes taken up by mitochondria in a ΔΨm-dependent manner [33,34]. In healthy cells with a high ΔΨm, both of these mitochondrial membrane-sensitive dyes aggregated in the mitochondrial matrix and showed a high fluorescence signal compared to the distribution and signal observed in the cells in which damage had eliminated the ΔΨm. *C. albicans* cells were treated with various concentrations of hep 25 at 37 °C for 90 min. The cells were then washed twice with PBS and stained with 50 nM DiOC6(3) or 100 nM TMRM at 37 °C for 30 min. The fluoresce intensity was analyzed using a flow cytometer (BD Accuri^®^ C6, BD Biosciences) with the FL2 filter.

### 2.8. Metacaspase Activity Assay

The activation of metacaspase was detected as previously described [35]. *C. albicans* cells were treated with various concentrations of hep 25 at 37 °C for 90 min, collected by centrifugation, and washed with PBS. The cells were then stained with 10 µM CaspACE^TM^ FITC-VAD-FMK in situ marker (Promega, Catalog #G746A, Madison, WI, USA) at RT for 30 min in the dark. Thereafter, the cells were harvested, washed twice, resuspended in PBS and analyzed using a flow cytometer (BD Accuri^®^ C6, BD Biosciences) with the FL1 filter.

### 2.9. Statistical Analysis

The data were assessed for statistical significance by two-tailed Student’s *t*-tests. Statistical significance was indicated with a *p*-value < 0.05.

## 3. Results

### 3.1. Hep 25 Shows Potent Candidacidal Activity in an Energy- and Temperature-Dependent Manner Without Causing Membrane Disruption

In this study, the activity of hep 25 (Table 1) was characterized.

**Figure 1:**
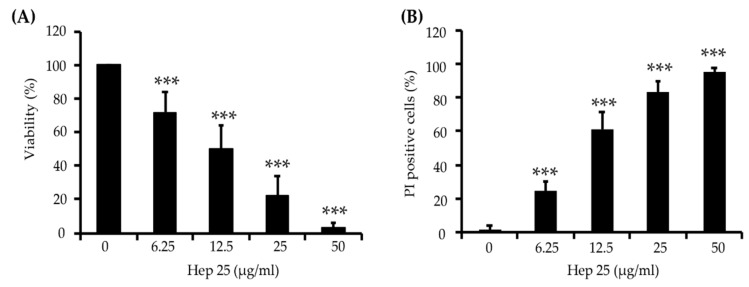
*C. albicans* killing assay of hep 25. (**A**) Killing of *C. albicans* by hep 25 was determined by the number of CFUs and expressed as the percentage of viable cells; *** *p* < 0.001. (**B**) The killing activity of hep 25 was detected by PI staining. The cells killed by hep 25 (PI-positive cells) were quantified by flow cytometry and reported as a percentage. The results are presented as the mean ± standard deviation of three independent experiments; *** *p* < 0.001.

To determine the efficiency of hep 25 against *C. albicans*, cell susceptibility assays were performed. As shown in Figure 1A, the viability of *C. albicans* cells after hep 25 treatment decreased with increasing concentrations of hep 25. Moreover, cell viability upon hep 25 treatment was also assessed using PI staining. Figure 1B shows that the number of inviable PI-positive cells increased with increasing concentrations of hep 25. To further verify the efficacy of hep 25, the susceptibilities of various clinical isolates of *C. albicans* and non-*albicans Candida* species (Table S1) to the peptide were also tested. Table S2 shows that the overall minimal fungicidal concentrations (MFCs) of hep 25 against non-*albicans Candida* spp. were lower than that of *C. albicans* isolates. Similarly, a sublethal dose (12.5 µg/mL) of hep 25 against the *C. albicans* isolates had a greater impact on cell survival of non-*albicans Candida* spp. (Figure S1). The results showed that hep 25 is not only effective against *C. albicans* but also non-*albicans Candida* spp.

**Figure 2:**
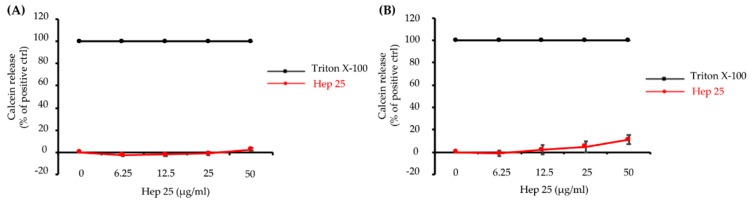
The effect of hep 25 on membrane integrity. Leakage of calcein from SUVs [PC:PE:PI:ergosterol = 5:4:1:2 (*w/w/w/w*)] was monitored after treatment with hep 25 for (**A**) 1 min and (**B**) 90 min. The induced calcein leakage was expressed as a percentage. Calcein leakage from the SUVs treated with 0.1% Triton X-100 was used as a positive control and defined as 100% leakage. The results are expressed as the mean ± standard deviation of three independent experiments.

The functions of AMP are generally either membrane targeting or nonmembrane targeting [7]. Because hep 25 causes *C. albicans* cell death, we were interested in determining whether hep 25 has membrane-disruption ability. Here, we detected the calcein leakage from SUVs that mimic the plasma membrane of *C. albicans* cells [28]. The calcein leaked from SUVs treated with 0.1% Triton X-100 treatment was used as a positive control and defined as having 100% leakage. Interestingly, no or negligible signals were detected in the SUVs treated with different concentrations of hep 25 after incubation for 1 min (Figure 2A) and 90 min (Figure 2B). These results suggest that hep 25-mediated *C. albicans* killing is independent of disrupting the membrane integrity.

**Figure 3:**
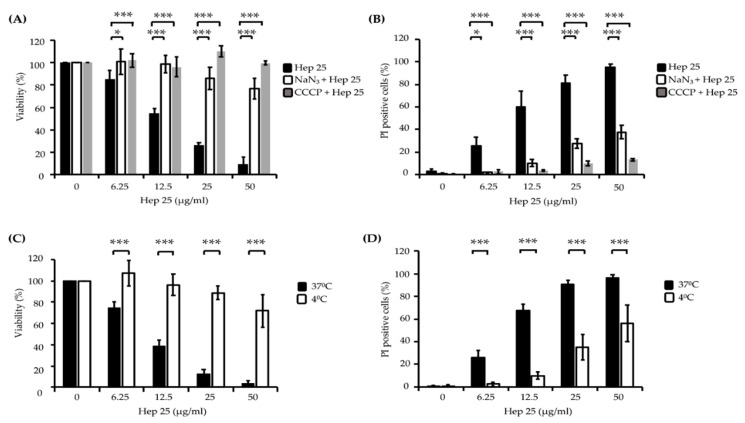
The effect of energy and temperature on the killing activity of hep 25. *C. albicans* cells were pretreated with 5 mM NaN_3_ for 1 h or 50 μM CCCP for 2 h at 30 °C to deplete cellular ATP, harvested by centrifugation, washed with sterile PBS and incubated with various concentrations of hep 25 at 37 °C for 90 min. (**A**) Cell viability was calculated by the number of CFUs and expressed as percentage with respect to the control cells (without hep 25 treatment). (**B**) Cells stained with PI were detected using a flow cytometer and expressed as percentages. *C. albicans* cells were treated with various concentrations of hep 25 at 4 °C for 90 min. (**C**) Candidacidal activity of hep 25 was determined by the number of CFUs and expressed as the percentage of viable cells. (**D**) The extent of hep 25-induced death as detected by PI staining. All the results are expressed as the mean ± standard deviation of three independent experiments; * *p* < 0.05 and *** *p* < 0.001.

It is known that some AMPs can enter cells without damaging the plasma membrane in an energy-dependent manner. For example, histatin 5 (Hst 5) gains access to the cytosol through transporters or receptor-mediated endocytosis [36,37,38], whereas pep-1 translocates through a physical-mediated mechanism [39]. To determine whether hep 25 translocation across the membrane requires energy consumption, cells were pretreated with the metabolic inhibitors sodium azide (NaN_3_) and carbonyl cyanide m-chlorophenyl hydrazine (CCCP) and then incubated with hep 25. NaN_3_ inhibited the mitochondrial complex IV, blocking mitochondrial respiration, whereas CCCP destroyed the proton gradient generated in mitochondria, blocking ATP synthesis [40]. The results indicated that both NaN_3_ and CCCP can rescue cells from hep 25 killing, as determined by the number of CFUs (Figure 3A) and PI staining evidence (Figure 3B). Low temperatures diminish cell metabolism and ATP synthesis, and moreover, they can be used to determine the entry of AMPs into cells by distinguishing translocation (occurs at 37 °C and 4 °C) from endocytosis (occurs only at 37 °C) [40,41,42,43]. Therefore, to further determine the method by which hep 25 gains access to *C. albicans*, cells were incubated with hep 25 at 4 °C and 37 °C, and cell viability was measured. The candidacidal activity of hep 25 was reduced at 4 °C compared to that at 37 °C, as demonstrated by the number of CFUs (Figure 3C) and the PI staining evidence (Figure 3D). Taken together, these findings suggest that endocytosis may be the process by which hep 25 is transported into *C. albicans* cells.

### 3.2. The ATUCN Motif of Hep 25 Is Involved in ROS Production and Correlates with the Activity of Hep 25

**Figure 4:**
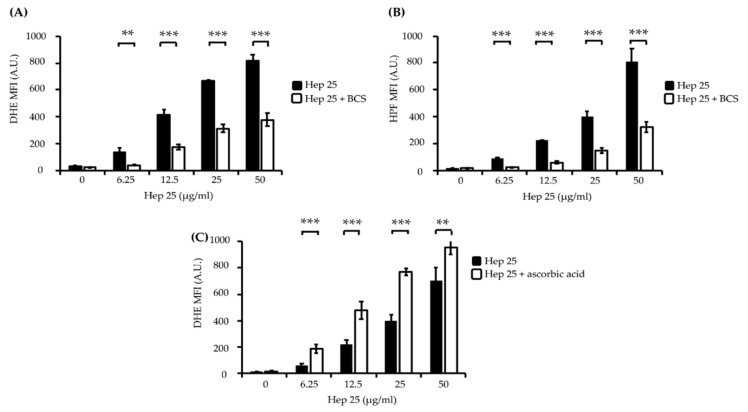
The induction of intracellular ROS production by hep 25. Intracellular ROS production was assessed through DHE (**A**,**C**) and HPF (**B**) staining and flow cytometry. The black bar and white bar show cells treated with hep 25 and cotreated with 1 mM BCS or 10 mM ascorbic acid and hep 25, respectively. MFI, mean fluorescence intensity and A.U., arbitrary unit. The results are presented as the mean ± standard deviation of three independent experiments; ** *p* < 0.01 and *** *p* < 0.001.

ROS play roles in the apoptosis of different cell types, and the induction of ROS production is also an important antimicrobial mechanism for many AMPs [44,45]. Several studies show that the ATCUN motif is related to the ability of certain AMPs to promote the production of ROS, particularly hydrogen peroxide and hydroxy radicals involving redox-active metals and a Fenton-like reaction [46,47,48,49,50]. DHE and HPF staining were used to detect hydrogen peroxide and hydroxyl radical levels, respectively. The fluorescence intensity of these ROS indicators was proportional to the ROS levels determined by the flow cytometry analysis [51,52]. As shown in Figure 4A,B, the fluorescence intensity of DHE and HPF was enhanced in the *C. albicans* cells treated with various concentrations of hep 25.

**Figure 5:**
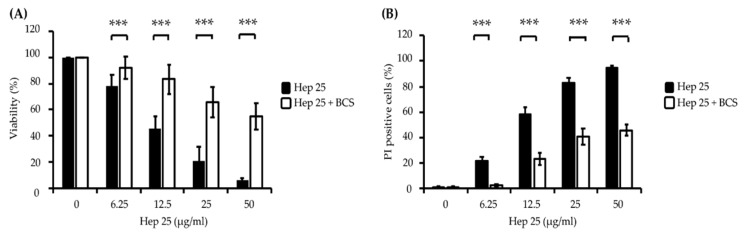
The effect of BCS on the candidacidal activity of hep 25. (**A**) Killing of *C. albicans* cells by hep 25 cotreated with or without 1 mM BCS was determined by the number of CFUs and expressed as the percentage of viable cells; *** *p* < 0.001. (**B**) The killing activity of hep 25 with or without 1 mM BCS was detected by PI staining. The cells killed by hep 25 with or without 1 mM BCS were quantified by flow cytometry and reported as a percentage. The results are presented as the mean ± standard deviation of three independent experiments; *** *p* < 0.001.

Bathocuproinedisulfonic acid (BCS) is a chelator known to block copper binding to the ATCUN motif-containing AMP histatin-5, leading to abrogated fungicidal activity of the peptide [50]. Interestingly, the fluorescence intensity of DHE and HPF in the cells cotreated with 1 mM BCS and hep 25 was significantly reduced (Figure 4A,B). Ascorbic acid (vitamin C) has been shown to act as a metal reducing agent to enhance the copper redox cycling of the Cu(II)-ATCUN motif, which is involved in ROS production through a Fenton-like reaction [53,54,55]. Therefore, to further verify the ROS production, cells were cotreated with ascorbic acid and hep 25. The results show that cells cotreated with hep 25 and ascorbic acid have a higher level of ROS production than that treated with hep 25 alone (Figure 4C). Moreover, the candidacidal activity of hep 25 was also reduced in the presence of BCS, as revealed by the number of CFUs and PI staining evidence (Figure 5A,B).

### 3.3. Hep 25 Induces Mitochondrial Depolarization in C. albicans

**Figure 6:**
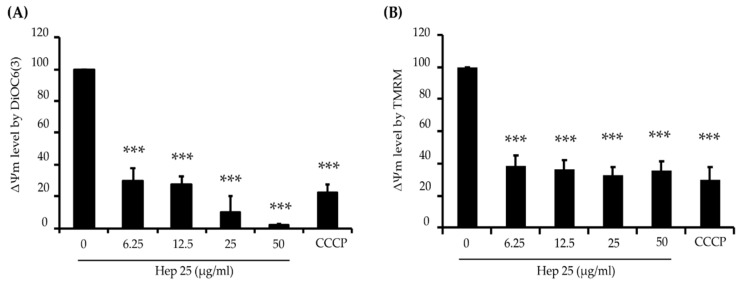
Mitochondrial depolarization caused by hep 25. Depolarization of the mitochondrial membrane was detected by DiOC6(3) (**A**) and TMRM (**B**) staining and analyzed using flow cytometry and quantification data. CCCP (50 μM) was used as a positive control for disruption of the mitochondrial membrane potential (ΔΨm). The results are expressed as the mean ± standard deviation of three independent experiments; *** *p* < 0.001.

Excessive ROS production causes oxidative stress, damages vital components of cells and induces cell death [56,57]. Because hep 25 does not seem to cause membrane disruption in *C. albicans* cells (Figure 2A,B), we were interested in the mechanism by which hep 25 causes cell death. Therefore, several hallmarks of apoptosis including mitochondrial depolarization, phosphatidylserine exposure and DNA fragmentation [58] were measured. To determine possible mitochondrial depolarization induced by hep 25, the lipophilic cell-permeant green-fluorescent dye DiOC6(3) was used. DiOC6(3) selectively detects the mitochondrial membrane potential (ΔΨm) of live cells [59]. Loss of ΔΨm is common during early apoptosis; therefore, mitochondrial depolarization is an indicator of mitochondrial-mediated apoptosis [60,61]. In this study, cells treated with the carbonyl cyanide m-chlorophenyl hydrazine (CCCP) and cells without treatment were used as the positive and negative controls, respectively. As shown in the flow cytometric analysis (Figure S2 and Figure 6A), the ΔΨm of the CCCP-treated cells was reduced compared to that of the negative control cells. Similar results were also found in cells treated with different concentrations of hep 25 (Figure S2 and Figure 6A). To verify this finding, another cell-permeant, the cationic fluorescent dye tetramethylrhodamine methyl ester (TMRM) was also used. TMRM accumulates in the highly negatively charged interior of mitochondria and its fluorescence intensity reflects the ΔΨm across the inner mitochondrial membrane [59]. A loss of ΔΨm results in the TMRM leakage from the mitochondria. As shown in Figure S3 and Figure 6B, the fluorescence intensity decreased in cells treated with hep 25, suggesting that hep 25 causes mitochondrial depolarization.

### 3.4. Hep 25 Triggers Metacaspase Activation

**Figure 7:**
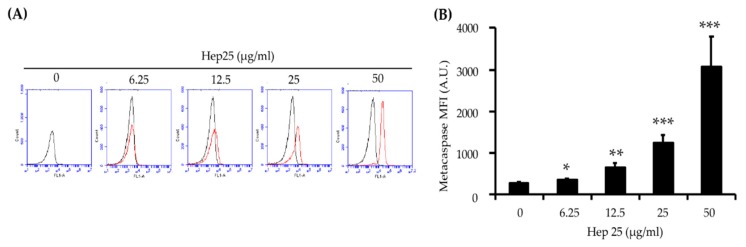
The activation of metacaspases by hep 25. Metacaspase activity induced by hep 25 was detected using the FITC-VAD-FMK in situ marker. In the flow cytometry data (**A**), the black line represents the cells without hep 25 treatment, which served as the negative control. The red line represents cells treated with various concentrations of hep 25. After treatment with hep 25, the cells were incubated with FITC-VAD-FMK for 20 min at RT in the dark and analyzed with a flow cytometer. The quantification data for (**B**). The results are expressed as the mean ± standard deviation of three independent experiments; * *p* < 0.05; ** *p* < 0.01; and *** *p* < 0.001.

Metacaspases are found in protozoa, fungi and plants, where they play important roles in regulating apoptosis [62]. Moreover, ROS accumulation and mitochondrial dysfunction are associated with metacaspase activation [63,64]. To verify that hep 25 is an apoptotic stimulus, metacaspase activity was measured using a CaspACE™ FITC-VAD-FMK in situ marker [65]. As shown in Figure 7A,B, an increase in FITC fluorescence intensity was detected with increasing concentrations of hep 25, indicating that hep 25 triggers metacaspase activation.

### 3.5. Phosphatidylserine was Externally Exposed in C. albicans Treated with Hep 25

**Figure 8:**
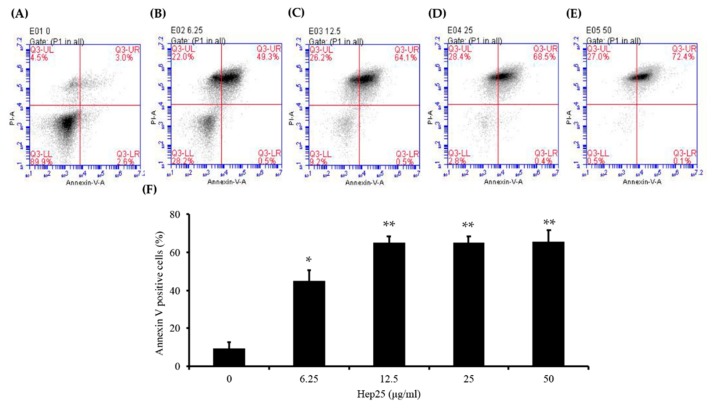
The induction of phosphatidylserine exposure by hep 25. After treatment with various concentrations of hep 25—(**A**) 0, (**B**) 6.25 μg/mL, (**C**) 12.5 μg/mL, (**D**) 25 μg/mL, and (**E**) 50 μg/mL—protoplasts were stained with Annexin V-FITC and PI and analyzed by flow cytometry. (**F**) The early and late apoptotic cells were stained with Annexin V-FITC (as shown in lower right quadrant, LR and higher right quadrant, UR). The percentage of apoptotic cells in total cell population was calculated. The data represent the mean ± standard deviation for three independent experiments; * *p* < 0.05; ** *p* < 0.01.

To further reveal the action of hep 25, cell death caused by hep 25 was also evaluated using Annexin V-FITC and PI staining. FITC-conjugated Annexin V specifically binds to PS, which is translocated to the outer leaflet of the plasma membrane during early apoptosis, staining the cell surface green. Moreover, after PS translocation, cells lose their plasma membrane integrity, allowing the red fluorescent PI dye to pass through and bind to nucleic acids. Therefore, cells stained with Annexin V-FITC are apoptotic [58,66]. Our results showed that the number of Annexin V-FITC positive cells increase after treatment with difference concentration of hep 25 compared to the control cells (without hep 25 treatment) (Figure 8A–F). These results suggest that hep 25 induces *C. albicans* apoptosis.

**Figure 9:**
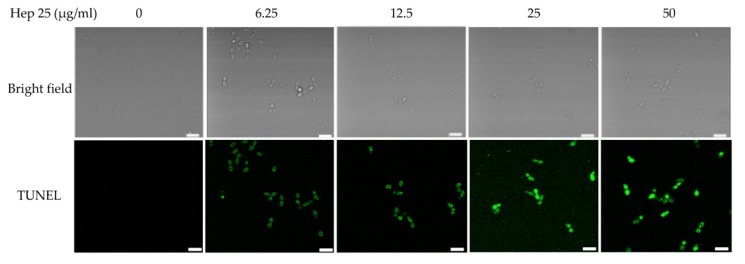
DNA fragmentation caused by hep 25. Cells were treated with various concentrations of hep 25, and the DNA damage was determined using the TUNEL assay. Images were taken using a laser scanning confocal microscope with a 60× oil objective lens. Bar, 10 μm. The representative images from three independent experiments with similar results are shown.

Finally, a FITC-dUTP nick-end labeling (TUNEL) assay [35,67,68] was also performed to detect DNA fragmentation in cells treated with hep 25. Figure 9 shows that there was no signal in the control cells; however green fluorescence was observed in the cells treated with hep 25. In summary, these results suggest that hep 25 induces *C. albicans* cells apoptosis.

## 4. Discussion

AMPs are potential candidates for development as new antifungal drugs. Hep 25 is a noncytotoxic human AMP with a broad spectrum of antibacterial activity [15]. In this study, we studied the mechanism by which hep 25 kills *C. albicans* and found that hep 25 killed *C. albicans* cells in an energy- and temperature-dependent manner, as determined by the results from the use of chemical inhibitors and reduced temperature conditions (Figure 3A–D). Nevertheless, we also found that nonfermentable glycerol, which forces cells to use mitochondrial oxidative phosphorylation to produce ATP, can enhance the candidacidal activity of hep 25 treated cells. Interestingly, hep 25 also exhibits nonmembrane lytic antimicrobial activity.

Of special interest is the effect of hep 25 on the specific binding of the ATCUN motif to Cu(II) and Ni(II) [69]. ATCUN-containing proteins exert various functions. For example, human serum albumin binding to Cu(II) is associated with metal transportation [70], whereas human protamine HP2 binding with metal is associated with damage to sperm DNA and ROS generation [71]. Moreover, adding an N-terminal ATCUN motif to different AMPs can enhance the activity of these peptides by inducing ROS formation [46]. Moreover, previous studies also indicated that the Cu(II)-ATCUN complex can damage DNA, RNA, proteins and lipids because it induces ROS generation [72,73,74]. Therefore, ROS are important for AMPs to killing of microbial pathogens [75,76,77,78] and are possibly generated through a Fenton-like reaction involving the Cu(II)-ATCUN complex [74,79]. Hence, we first determined the metal dependence of hep 25 on ROS production and killing activity by cotreating cells with the Cu chelator BCS. Our results indicated that 1mM BCS not only abrogates ROS production, but also reduces the *C. albicans* killing activity induced by hep 25 (Figure 4 and Figure 5). To further verify the effect of copper chelation on the candidacidal activity of hep 25, 2 mM BCS was also used [50]. Interestingly, the activity of hep 25 was almost completely inhibited by 2 mM BCS (Figure S4). Taken together, our findings show that ROS generation is crucial to hep 25 fungicidal activity, and the primary source of ROS is derived from the Cu(II)-ATCUN complex. However, some questions still need to be addressed: For example, how efficiently does BCS compete with hep 25 for copper binding? Does hep 25 acquire copper extracellularly or intracellularly? Does hep 25 impair metal homeostasis?

Hep 25 does not appear to target the membrane (Figure 2), and it induces ROS generation (Figure 4A,B). Because ROS are associated with yeast apoptosis in many cases [80], it was interested in determining whether hep 25 induces *C. albicans* cell death via apoptosis. In the early phase of apoptosis, the opening of the permeability transition pore causes mitochondrial dysfunction, losses of ΔΨm and releases of apoptogenic factors into the cytosol [81,82]. Moreover, metacaspases are involved in the apoptotic cascade [83,84]. Our results showed that hep25 causes mitochondrial depolarization (Figure 6) and induces metacaspase activation (Figure 7). Finally, Annexin V-FITC and PI double staining and TUNEL assays revealed PS exposure and DNA fragmentation (Figure 8 and Figure 9), which are all important features of apoptosis.

In summary, we demonstrated that hep 25 induces *C. albicans* apoptosis without disrupting plasma membrane integrity. Moreover, the ATCUN motif of hep 25 is important for the candidacidal activity of the peptide. Therefore, our findings provide important information on how an ATCUN-containing peptide can kill fungal pathogens and demonstrate its potential for future use in antifungal therapy.

## Figures and Tables

**Table 1:** Amino acid sequence and physicochemical properties of Hep25.

Peptide	Amino Acid Sequence	Disulfide Bonds	Molecular Mass (Da)	Net Charge ^a^	Hydrophobicity ^a^
Hep 25	DTHFPICIFCCGCCHRSKCGMCCKT	7–23, 10–13, 11–19,14–22	2789.4	2	52%

^a^ Net charge and Hydrophobic ratio were calculated using the Antimicrobial Peptide Calculator and Predictor (http://aps.unmc.edu/AP/prediction/prediction_main.php).

## Supplementary Materials

The following are available online at https://www.mdpi.com/2076-2607/8/4/585/s1, Figure S1: The activity of hep 25 against various clinical isolates of *C. albicans* and non-*albicans Candida* spp. Figure S2: Mitochondrial depolarization detected with DiOC6(3). Figure S3: Mitochondrial depolarization detected by TMRM. Figure S4. The effect of 2 mM BCS on the candidacidal activity of hep 25. Table S1: Clinical isolates of *Candida* spp. used in this study. Table S2: Minimal fungicidal concentrations (MFCs) of hep 25 against *C*. *albicans* and non-*albicans Candida* clinical isolates.
