# Supplementary material for: Human Antimicrobial Peptide Hepcidin 25-Induced Apoptosis in Candida albicans
Source: Microorganisms. 2020 Apr 17;8(4):585. doi: 10.3390/microorganisms8040585 (PMC7232333; doi:10.3390/microorganisms8040585)
Supplement: Supplementary file 1 [file microorganisms-08-00585-s001.pdf]

Table S1. Clinical isolates of *Candida* spp. used in this study.

Candida spp.	**Source**[a]	**Strain**[b]
C. albicans	ATCC MYA-2876	SC5314
C. albicans	ATCC90028	YLO12
C. albicans	HIV patient	YH050001
C. albicans	HIV patient	YH050005
C. albicans	HIV patient	YH050072
C. glabrata	ATCC9003	YLO8
C. glabrata	HIV patient	YH050105
C. krusei	ATCC6258	YLO6
C. krusei	HIV patient	YH050075
C. tropicalis	ATCC13803	YLO86
C. tropicalis	HIV patient	YH050007
C. tropicalis	HIV patient	YH050013
C. tropicalis	HIV patient	YH050114
C. parapsilosis	ATCC22019	YLO7
C. dubliniensis	HIV patient	YH050092

[a] HIV patient, *Candida* strains isolated from HIV-infected patients.

[b] These strains were provided by Hsiu-Jung Lo (Yu et al., Antimicrob Agents Chemother. 2011, 55:4918-4921).

Table S2. Minimal fungicidal concentrations (MFCs) of hep 25 against *C. albicans* and non-*albicans Candida* clinical isolates.

Candida spp.	**Source**	**Strain**	**MFCs (μg/ml)**[a]
C. albicans	ATCC MYA-2876	SC5314	100
C. albicans	ATCC90028	YLO12	>50
C. albicans	HIV patient	YH050001	>50
C. albicans	HIV patient	YH050005	>50
C. albicans	HIV patient	YH050072	>50
C. glabrata	ATCC9003	YLO8	50
C. glabrata	HIV patient	YH050105	50
C. krusei	ATCC6258	YLO6	25
C. krusei	HIV patient	YH050075	25
C. tropicalis	ATCC13803	YLO86	25
C. tropicalis	HIV patient	YH050007	25
C. tropicalis	HIV patient	YH050013	25
C. tropicalis	HIV patient	YH050114	50
C. parapsilosis	ATCC22019	YLO7	25
C. dubliniensis	HIV patient	YH050092	50

[a]The MFCs were defined as the lowest drug concentration that killed ≥ 99.9% of cells and determined according to Basso et al. (Antimicrob Agents Chemother. 2018, 62(6)) with some modifications.

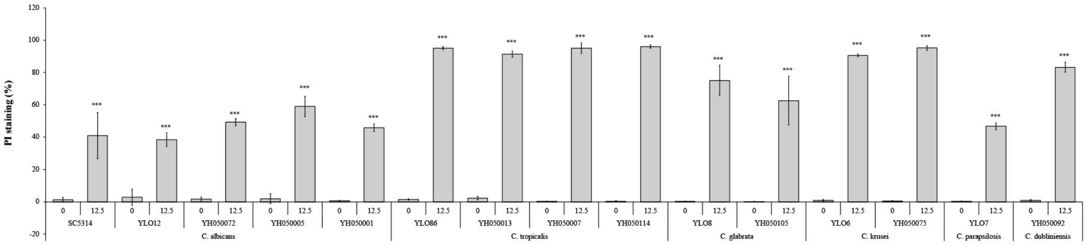

Figure S1. The activity of hep 25 against various clinical isolates of *C. albicans* and non-*albicans Candida* spp. The cells killed by hep 25 (PI-positive cells) were quantified by flow cytometry and normalized to the number of control cells (without hep 25 treatment) and reported as a percentage. 0: cells without hep 25 treatment, 12.5: cells with 12.5 μg/ml hep 25 treatment. The results are presented as the mean ± standard deviation of three independent experiments; *** $P < 0.001$.

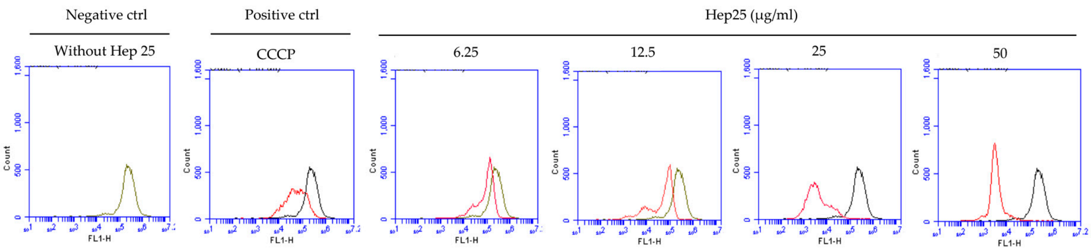

Figure S2. Mitochondrial depolarization detected with DiOC6(3). Cells treated with various concentrations of hep 25 or with 50 μM of CCCP (red line) had decreased MMP fluorescence intensity compared to that of the untreated cells (black line). The results shown are from one of the three independent experiments.

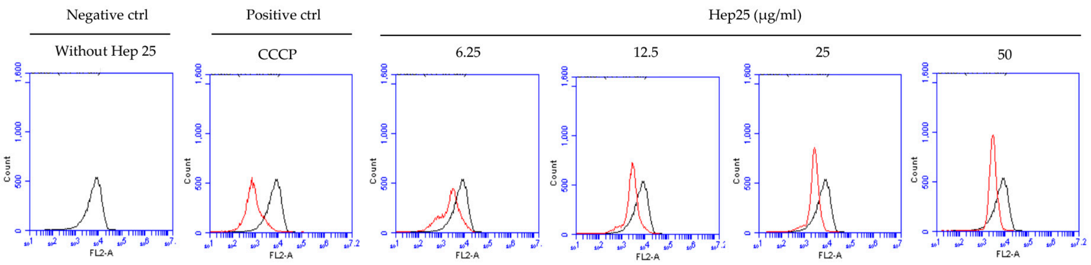

Figure S3. Mitochondrial depolarization detected by TMRM. Cells treated with various concentrations of hep 25 or with 50 μM CCCP (red line) had a decreased MMP fluorescence intensity compared to that of the untreated cells (black line). The results shown are from one of the three independent experiment.

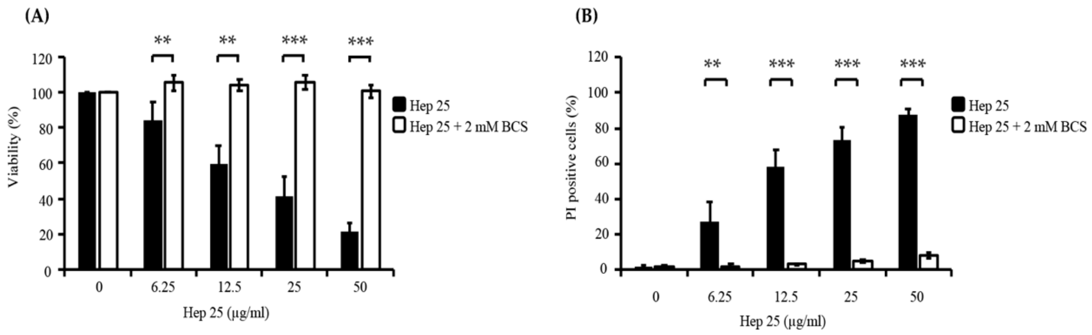

Figure S4. The effect of 2mM BCS on the candidacidal activity of hep 25. (A) Killing of C. albicans cells by hep 25 cotreated with or without 2 mM BCS was determined by the number of CFUs and expressed as the percentage of viable cells; ** P <0.01 and *** P < 0.001. (B) The killing activity of hep 25 with or without 2 mM BCS was detected by PI staining. The cells killed by hep 25 with or without 2 mM BCS were quantified by flow cytometry and reported as a percentage. The results are presented as the mean ± standard deviation of three independent experiments; ** P <0.01 and *** P < 0.001.
